# Towards Sustainable Bone Grafting: Life Cycle Assessment of Donor Cadaver-Derived Allograft (BMG) Production Using a BMP-Preserving Approach

**DOI:** 10.3390/jfb17040171

**Published:** 2026-04-01

**Authors:** Szidonia Krisztina Veress, Mihai Vlad Golu, Lajos Csönge, Bernadette Kerekes-Máthé, Melinda Székely, Bálint Botond Bögözi

**Affiliations:** 1 Doctoral School of Medicine and Pharmacy, IOSUD, George Emil Palade University of Medicine, Pharmacy, Science, and Technology of Targu Mures, 540142 Targu Mures, Romania; szidonia-krisztina.veress@umfst.ro; 2 Department of Oral and Maxilo-Facial Surgery, Faculty of Dentistry, George Emil Palade University of Medicine, Pharmacy, Science, and Technology of Targu Mures, 38 Gheorghe Marinescu Str., 540142 Targu Mures, Romania; balint.bogozi@umfst.ro; 3 West-Hungarian Regional Tissue Bank, Petz Aladár University Teaching Hospital, Vasvári P. u. 2-4., 9024 Győr, Hungary; luisbathhelena@gmail.com; 4 Hisztolabor Kft, Kócsag u.2, 9026 Győr, Hungary; 5 Department of Teeth and Dental Arches Morphology, Faculty of Dentistry, George Emil Palade University of Medicine, Pharmacy, Science, and Technology of Targu Mures, 38 Gheorghe Marinescu Str., 540142 Targu Mures, Romania; bernadette.kerekes-mathe@umfst.ro (B.K.-M.); melinda.szekely@umfst.ro (M.S.)

**Keywords:** allografts, bone grafting, dental implantation, orthopedic surgery, bone morphogenetic protein, sustainable development, disability-adjusted life years

## Abstract

Background/Objectives: Healthcare activities contribute significantly to climate change and environmental pollution. The demand for bone grafting is increasing, and the biological properties of bone substitute materials are critically important. A methodology aimed at preserving BMPs may offer an opportunity to improve the biological properties of donor cadaver-derived bone grafts. The aim of this study was to conduct a life cycle assessment of the BMP-preserving approach used in allograft production in order to enhance the environmental sustainability of bone grafting. Methods: Following primary data collection at the West Hungarian Regional Tissue Bank, environmental impacts were assessed using the OpenLCA software and the ReCiPe v1.03 (2016) midpoint and endpoint impact categories. A sensitivity analysis was also conducted under six alternative scenarios to evaluate which changes would have the greatest beneficial effect on environmental impacts. Results: The greatest environmental impacts of allograft production were observed in the categories of material resources: metals and minerals, terrestrial ecotoxicity, and climate change. The climate change impact was 66.759 kg CO_2_-eq. The environmental impacts of the production process also had a significant influence on human health, with a total DALY value of 6.58 h. The impacts were primarily driven by electricity consumption and the chemicals used; however, in several impact categories, waste management also contributed substantially. Conclusions: Transitioning to more sustainable energy sources (e.g., wind power) would substantially improve the environmental performance of allograft production. Further research is needed to identify more sustainable alternatives for the chemical agents used during processing.

## 1. Introduction

Bone tissue is the second most commonly transplanted tissue after blood transfusions [1,2,3,4]. Bone grafting is often used in the fields of oral surgery, orthopedics, and traumatology to treat bone defects caused by diseases, surgeries, trauma, or congenital anomalies [5]. In addition to their supportive and protective roles, bones play an important part in various vital physiological processes, including hematopoiesis and the storage of various minerals and growth factors. Bone loss can make a person disabled not only physically but also mentally and economically [2,5,6].

There are numerous bone substitute materials and surgical techniques available for bone replacement, and the success of the procedure, as well as the speed of recovery, are fundamentally determined by the type of bone substitute material used [3,7]. For the successful integration of a graft, its osteoconductive, osteoinductive, and osteogenic properties are essential [8,9]. In addition, the optimal bone substitute material should be easily and sufficiently available, cost-effective, and free from the risk of infection [3,10].

Based on its biological properties, autologous bone can be considered the gold standard for bone replacement; however, it has several disadvantages, including donor site morbidity, limited availability, and the need for additional surgical intervention [1,5,7]. An alternative to autografts is allograft bone, which, due to its human origin, possesses many of the same properties as autografts, such as osteoconductive and osteoinductive effects and biocompatibility [3,11,12]. In addition, it is available in larger quantities, can be provided in the desired form, and its use reduces surgical time [7,11]. Allografts can induce bone formation through the action of bone morphogenetic proteins (BMP) [13]. During allograft production, a targeted processing step is required to ensure the bioavailability of BMPs [14].

Bone grafting constitutes a substantial component of healthcare practice, and in aging societies, the demand for grafts used to replace bone defects is steadily increasing [5,11,12]. Regenerative medicine and tissue engineering have gained increasing attention in response to the growing demand for bone augmentation and the need to improve quality of life [15].

Due to the increasing demand for allografts, Tissue Banks have been established to provide allografts from living donors and cadavers. Tissue Banks ensure the collection, processing, and distribution of transplantable human tissues while adhering to strict standards and quality control measures [5,16,17]. Human freeze-dried cortical bone matrix gelatin (BMG), a product of the West Hungarian Regional Tissue Bank in Győr (WHRTB), has been used for more than three decades with excellent outcomes in both orthopedic and oral surgery [18,19].

Climate change is one of the most significant challenges to public health. Healthcare systems, while their primary goal is to preserve health, currently contribute substantially to greenhouse gas (GHG) emissions and environmental pollution [20,21,22]. The healthcare sector is responsible for approximately 4.0–8.5% of global GHG emissions; thus, if healthcare were considered a country, it would be the fifth-largest GHG emitter worldwide [23,24,25]. The health impacts of climate change will further increase the burden on healthcare systems, thereby intensifying the need to adopt sustainable practices in healthcare [26,27,28].

There is growing environmental awareness among healthcare professionals [29]. Efforts are needed to increase awareness, expand knowledge, generate clinically relevant evidence to inform changes in established practices, and support decision-making in order to achieve environmentally sustainable healthcare practices [29,30].

Life cycle analysis (LCA) is an analytical tool used to assess the environmental impacts of products or processes and to evaluate their potential effects on human health, thereby supporting sustainability-related decision-making [20,23,29,31].

Climate change increases the risk of infectious diseases as well as conditions related to heat, pollution, and natural disasters (e.g., cardiovascular diseases, asthma, malaria, and diarrhoeal diseases), which disproportionately affect vulnerable populations [30,32,33]. Disability-adjusted life years (DALYs) are a key metric used in public health assessments. In life cycle assessment (LCA), DALYs are used to standardize the potential impacts of environmental pressures on human health, commonly through the ReCiPe method [23].

HealthcareLCA is an online database that compiles life cycle analyses of healthcare-related activities. At present, the database does not include life cycle analyses related to bone grafting in implantology, oral surgery, or orthopedics, nor analyses addressing the production of allografts or other bone grafting materials [20,34]. In addition, the environmental impacts associated with tissue bank activities themselves have received very limited attention in the sustainability literature. Furthermore, many life cycle assessment studies in the healthcare and dental care sectors report results primarily at the midpoint level, while endpoint indicators such as disability-adjusted life years (DALYs) are often not included. This represents a methodological limitation, as the absence of DALY-based results may restrict the interpretation of potential impacts on human health [23].

The aim of this study is to assess the life cycle of cadaver-derived allografts processed using a bone morphogenetic protein-preservation-oriented approach, and to present the results using both midpoint and endpoint impact categories. In addition, the study seeks to explore alternative scenarios involving key process components to evaluate the extent to which variations in these elements may influence the overall environmental impacts. More broadly, this work aims to initiate and support research on bone substitutes and bone grafting from an environmental sustainability perspective, with the goal of fostering environmentally conscious decision-making in bone grafting practices, alongside functional and clinical considerations.

## 2. Materials and Methods

The present study was approved by the Legal and Ethics Committee of Petz Aladár University Teaching Hospital (Approval no. 9/485-1/2025).

Life cycle assessment (LCA) is considered the gold standard for evaluating the sustainability and environmental impacts of products or processes. The method can also be applied to assess the environmental aspects of healthcare activities [22,23].

The International Organization for Standardization (ISO) 14040:2006 standard [23] and its 2020 amendment provide the methodological framework for implementing LCA. Accordingly, life cycle assessment can be divided into four main phases:

1. Definition of the goal and scope, including the functional unit and system boundaries
2. Compilation and evaluation of the life cycle inventory (LCI) using available databases
3. Life cycle impact assessment (LCIA), including midpoint and endpoint impact categories
4. Interpretation and evaluation of the results

### 2.1. Objective and Scope

#### 2.1.1. Goal, Scope

The aim of this study was to analyze the life cycle of the production of donor cadaver-derived allografts processed using a specialized methodology designed to preserve bone morphogenetic proteins (BMPs). As the study focuses exclusively on the manufacturing phase, a cradle-to-gate approach was applied.

#### 2.1.2. Functional Unit

The functional unit defines the specific product or process for which the life cycle assessment is performed. In this study, the functional unit was defined as a process of BMP-containing granular allograft produced from a donor cadaver and packaged in 1 g individual units for clinical use. This functional unit reflects the manufacturing output and enables the quantification of environmental impacts associated with the production process itself. It should be emphasized that the functional unit does not imply clinical or biological equivalence with other bone grafting materials, such as xenografts or synthetic substitutes, as differences in osteoinductive potential, biological activity, and clinical indication may result in different material requirements in practice. Therefore, the present life cycle assessment focuses on the environmental performance of the manufacturing process per unit mass, rather than on a direct functional comparison between different graft materials.

#### 2.1.3. System Boundaries

In life cycle assessment, system boundaries define the processes and flows considered within the analysis. They include the inputs, processes, and outputs or waste streams associated with the functional unit.

**Figure 1:**
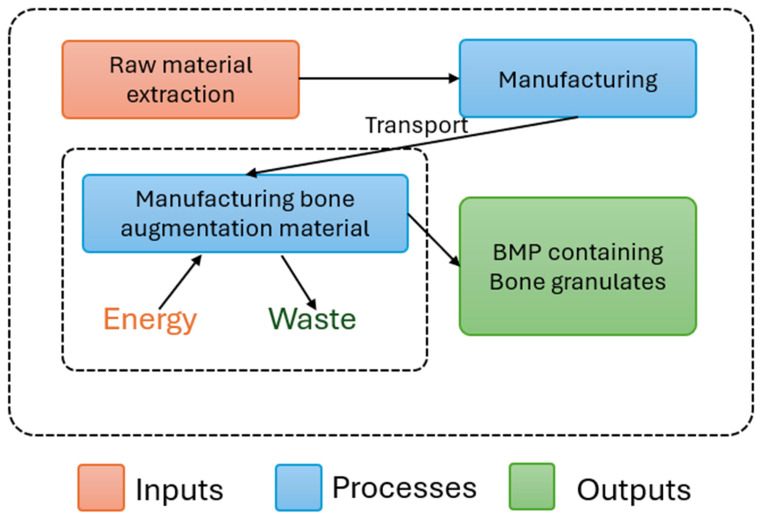
System boundaries.

System boundaries can be defined as cradle-to-grave, when the entire life cycle of a product is assessed from production to disposal, or cradle-to-gate, when the analysis is limited to the production phase. In this study, a cradle-to-gate approach was applied, thereby enabling comparison with other manufacturing processes. Accordingly, the system boundaries included the chemicals used during allograft production, electricity consumption, disposable materials, final product packaging, and waste management. The defined system boundaries are illustrated in Figure 1.

The following were not included within the system boundaries:

1. Downstream processes were not included. The clinical use of allografts may vary, and their follow-up and standardization would be methodologically uncertain; however, the results obtained in this study may serve as input data for future life cycle assessments of surgical procedures involving allograft-based bone substitutes.
2. The tools used during processing (e.g., glass containers for chemical solutions and forceps used during packaging) were excluded from the system boundaries, as they are used minimally during the process, repeatedly reused, and therefore assumed to have a negligible environmental impact.
3. Device maintenance was also excluded from the system boundaries, as maintenance activities occur infrequently throughout the production process and are therefore assumed to have a negligible impact.
4. The activities of Tissue Bank staff were not included within the system boundaries, as their tasks are diverse, cannot be attributed exclusively to the production of cadaver-derived allografts, and the individual production steps are temporally separated, making allocation methodologically undefined and uncertain.
5. Serological testing of donors was also excluded from the system boundaries, as it is conducted outside the Tissue Bank’s activities and is required for the transplantation of all donor tissues; therefore, its allocation specifically to bone graft production is associated with a high degree of uncertainty.

### 2.2. Analysis of the Life Cycle Inventory

Primary data were collected at the West Hungarian Regional Tissue Bank within the framework of the Petz Aladár University Teaching Hospital. Quantification of materials and equipment was based on systematic observations and direct measurements. The assumptions used in the analysis were derived from empirical observations and the Tissue Bank’s accumulated professional experience.

The following assumptions were applied in the calculations:

1. In one processing cycle, the diaphysis of long tubular bones (humerus, radius, ulna, femur, tibia, fibula) from one donor cadaver is processed.
2. All procedures are performed by a single operator wearing full personal protective equipment, including a sterile surgical gown, surgical mask, hair cover, and shoe covers.
3. Prior to processing, the bones are stored in a freezer for an average of 28 days.
4. Residual soft tissue is removed from the bones during the procedure using a bone-cleaning machine.
5. The epiphyses are removed from the bones, and the cortical bone is sectioned to facilitate the degreasing process.
6. The degreasing process is carried out in a methanol–chloroform solution and lasts an average of 21 days. The solution is initially replaced every 2 days and subsequently every 3 days. After completion of the degreasing process, the bones are ground.
7. Waste generated during bone grinding is treated as infectious waste, as it becomes unsuitable for further use as an allograft due to contamination with metal particles.
8. The ground bone is subjected to a specialized processing methodology and treated with various chemical solutions (HCl, CaCl_2_, EDTA, LiCl, H_2_O_2_) with the aim of preserving bone morphogenetic proteins (BMPs) in the bone substitute. Between solution changes, the bone is rinsed with deionized water. This BMP-preserving processing method differs from conventional allograft techniques by including the demineralization step to release BMPs, followed by remineralization to restore the bone matrix.
9. Bone granules are sieved using a sieving machine and packaged separately according to particle size.
10. The bone substitutes are sterilized using ethylene oxide at 50 °C, which, unlike gamma irradiation, does not degrade BMPs in the bone substitute.
11. Allografts are handled under sterile conditions on sterile fields throughout the processing steps.
12. The sterilized bone substitutes are lyophilized to enable long-term storage at room temperature. The lyophilization process lasts 24 h.
13. The lyophilized bone substitutes are individually packaged in 1 g packages under sterile conditions in a laminar airflow cabinet and subsequently double-wrapped to ensure appropriate use during surgery.
14. Packaging materials are sterilized separately in advance using an autoclave.

#### Life Cycle Inventory

During the preparation of the life cycle inventory, all steps of allograft production were thoroughly assessed to evaluate the environmental impacts of the process. The production steps are as follows: receipt of bones, storage in the freezer, cleaning and cutting, defatting, bone grinding, deantigenization and BMP-preservation chemical treatment, sieving of bone, size fractionation, ethylene oxide sterilization, lyophilization, and packaging.

Energy consumption for all steps was recorded. Device energy consumption was determined based on equipment performance and operating time (Table 1):

Disposable materials used in the process were disaggregated into their components, and their exact weights were measured using an analytical balance. To improve accuracy, 10 samples of each material were weighed. Information on material composition was obtained from product packaging and the manufacturer’s website.

Waste generated during production was categorized as follows: bone waste from cutting and grinding, and disposable materials that contacted bone, were classified as infectious waste; chemical substances were defined as hazardous chemical waste; and product packaging was classified as municipal solid waste. The hospital was responsible for the collection, storage, transportation, and disposal of all waste.

The life cycle inventory data used in this study are provided in Supplementary Material S1.

### 2.3. Assessing the Life Cycle Impact

To assess the environmental impacts of the allograft manufacturing process, a life cycle assessment (LCA) was conducted using OpenLCA v2.5.0. software. The analysis was based on a merged database comprising bioenergiedat_18; elcd_3_2_greendelta_v2_18_correction; needs_18; OzLCI2019; worldsteel_2020; EXIOBASE 3.9.4; USDA_1901009; and BAFU-2025_LCI DB_17Dec25.

Environmental impacts were quantified using the ReCiPe v1.03 (2016) midpoint (H) method, which evaluates life cycle impacts across 18 environmental impact categories. In addition, the ReCiPe v1.03 (2016) endpoint (H) method was applied to calculate disability-adjusted life years (DALYs) as an indicator of potential human health impacts.

To assess uncertainty, lognormally distributed uncertainty factors were assigned to the inputs contributing most to the environmental impacts (chemicals used, electricity, and final product packaging), and 5000 Monte Carlo iterations were subsequently performed.

A sensitivity analysis was performed to evaluate the robustness of the life cycle assessment results and to identify the key drivers of environmental impacts. The analysis examined the relative changes in the 18 ReCiPe midpoint impact categories and in the endpoint category expressed as disability-adjusted life years (DALYs) under six alternative scenarios compared to the baseline case. The scenarios considered were: (1) conventional chemical use, (2) reduced chemical use (−20%), (3) energy supplied from wind power, (4) reduced energy demand (−20%), (5) energy recovery from waste incineration, and (6) use of 5 g packaging units.

## 3. Results

This section presents the results of the life cycle assessment of the BMP containing cadaver-derived allograft (BMG) production analyzed in the study. The process results in 300 g of bone graft material.

**Figure 2:**
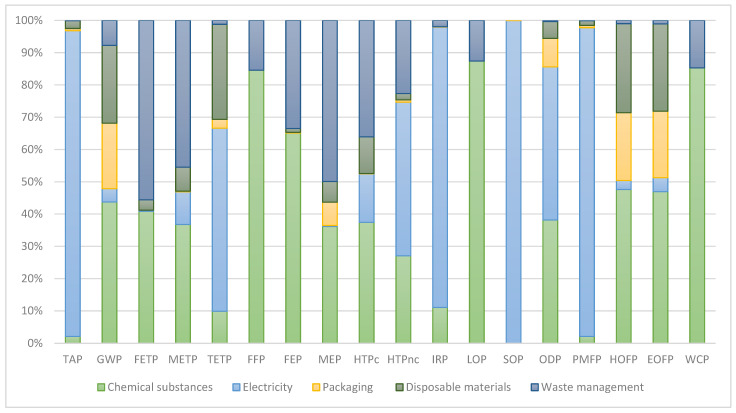
Breakdown of contribution to environmental impacts in 18 impact categories—relative role of individual factors (chemical substances, electricity, packaging of the final product, disposable materials and waste management).

Table 2 contains the results of the LCIA analysis in the ReCiPe v1.03 Midpoint (H) impact categories. In Figure 2, the relative contributions of key factors to environmental impacts are summarized.

The Results of the Monte Carlo simulations can be found in Supplementary Material S2. The Monte Carlo simulation results indicate that the LCA outcomes are relatively stable across several impact categories, with moderate differences between the mean, median, and percentile values, and low standard deviations in many cases. These findings suggest that the model exhibits a satisfactory level of robustness and that the results are not highly sensitive to variations in input parameters.

Table 3 contains the DALY values determined by the ReCiPe v1.03 2016 endpoint (H) method for the environmental impacts of one manufacturing process.

**Figure 3:**
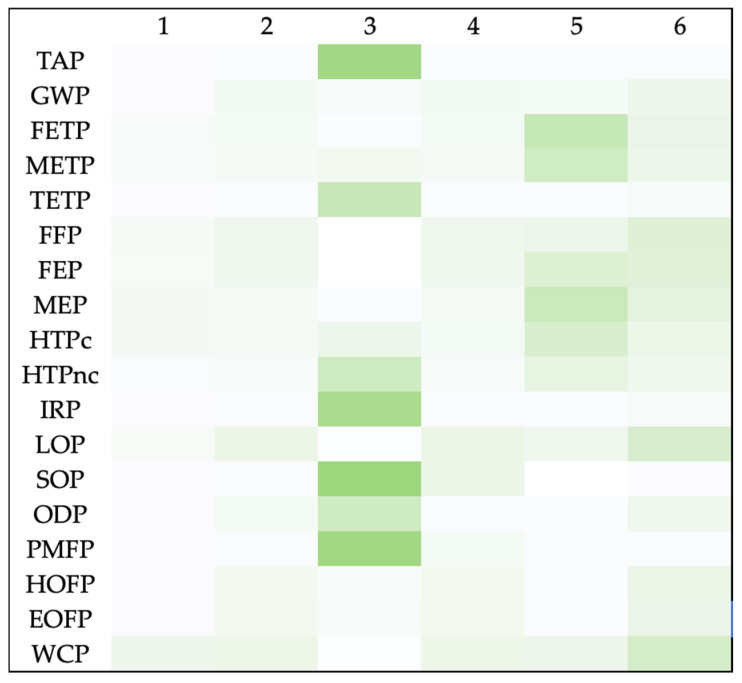
Heat map-based sensitivity analysis illustrating relative changes across 18 environmental impact categories under six alternative scenarios: (1) conventional chemical use, (2) reduced chemical use (−20%), (3) energy supplied from wind power, (4) reduced energy demand (−20%), (5) energy recovery from waste incineration, and (6) use of 5 g packaging units.

**Figure 4:**
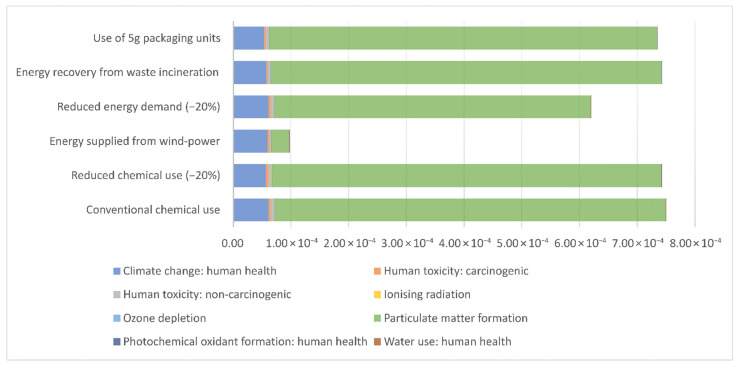
Sensitivity analysis of endpoint impacts expressed as disability-adjusted life years (DALYs) across 6 alternative scenarios. The chart presents the DALY contributions across the ReCiPe human health endpoint categories. Each category is represented by grouped bars for the scenarios: (1) conventional chemical use, (2) reduced chemical use (−20%), (3) energy supplied from wind power, (4) reduced energy demand (−20%), (5) energy recovery from waste incineration, and (6) use of 5 g packaging units.

Figure 3 and Figure 4 presents the results of sensitivity analysis. Detailed results of the sensitivity analysis in both midpoint and endpoint categories are provided in Supplementary Material S2.

The scenarios modeled in the sensitivity analysis were primarily intended to illustrate how environmental impacts may be influenced, both in direction and magnitude, by changes in the selected parameters. These scenarios should therefore not be interpreted as fully independent or comprehensive real-world cases. More precise quantification would require broader empirical data, additional measurements, and further research. Nevertheless, the results provide valuable insights and indicate potential directions for improving the environmental performance of the allograft production process.

## 4. Discussion

Bone is continuously remodeled to maintain its structure and function, making it a dynamic tissue characterized by its capacity for self-renewal [6]. It is a specialized connective tissue composed of cellular elements and a mineralized extracellular matrix. The cellular elements include osteoprogenitor cells, osteoblasts, osteocytes, and osteoclasts. The bone matrix is composed of organic and inorganic components, with 65–75% being inorganic, primarily hydroxyapatite and calcium- and phosphate-rich compounds [6,10,11,15]. The scaffold provides the extracellular microenvironment together with appropriate biological triggers that support cell-driven regeneration. However, bone healing may be impaired, and bone loss resulting from trauma, inflammation, or cancer can necessitate bone augmentation interventions [35].

Regeneration can be defined as the reproduction or re-formation of organs or tissues [13]. Effective bone regeneration requires several essential elements, including a biologically supportive and mechanically stable environment, osteoprogenitor cells, and the presence of various growth factors [14,36]. Bone grafts are tissues implanted into defects to promote and enhance bone healing [37,38]. An ideal bone graft material should stimulate osteoregeneration through three key mechanisms: osteoconduction, osteoinduction, and osteogenesis [10,11,14,38]. In addition, it should exhibit the following properties: ease of handling and shaping, cost-effectiveness, ready availability in sufficient quantities, biocompatibility, non-toxicity, lack of infection risk, absence of ankylosis, adequate mechanical strength and similarity to native bone, and minimal surgical intervention requirements [10,11,13,35,38].

Osteoconduction is the property of a bone substitute material to provide a structural scaffold that facilitates the migration, attachment, and growth of host osteogenic cells, thereby enabling bone growth on its surface [6,10,13,36,37].

Osteoinduction is the process by which growth factors in bone substitute materials—such as BMPs, PDGFs, VEGFs, and FGFs—stimulate mesenchymal stem cells to differentiate into osteoblasts and promote bone formation [10,13,36,39].

Osteogenicity is the ability to form new bone through cells present in the graft that are capable of differentiation into bone-forming cells [10,13,36,38].

Bone grafting is commonly required in implantology for the regeneration of the alveolar ridge, as well as in orthopedic surgery [40,41]. The choice of bone graft material remains widely debated, with opinions often differing among authors [42]. Traditionally, bone graft materials are derived from four sources: the patient’s own tissue (autograft), living or cadaveric human donors (allograft), animal donors (xenograft), and synthetic materials (alloplast) [11].

Autogenous bone grafts are considered the gold standard for bone augmentation because they possess osteoconductive, osteoinductive, and osteogenic properties [3,8,43,44]. Autografts can be obtained from a variety of donor sites and harvested from intraoral or extraoral locations using different techniques [11,45]. In oral implantology, intramembranous bone grafts (e.g., intraoral and calvarial) exhibit lower resorption rates compared to endochondral bone grafts (e.g., iliac bones, ribs) [44,45]. The disadvantages of autografts include donor site morbidity and pain associated with harvesting, as well as the limited availability of suitable bone [8,11,40].

An allograft is tissue obtained from one individual and transplanted into another member of the same species [10,40,46]. Because bone is not dependent on Rh factor (Rh) or human leukocyte antigen (HLA) matching, a graft from a healthy donor can be transplanted into another person without risk of rejection [5]. Allografts demonstrate a success rate comparable to that of autografts [11,47]. Their main disadvantage is the potential for disease transmission, which necessitates processing and storage techniques designed to minimize this risk and prolong shelf life [3,38,42]. Allografts possess osteoconductive properties, may exhibit osteoinductive potential in the presence of BMPs, and fresh-frozen allografts can retain osteogenic effects [36,38,48]. Allografts are processed in Tissue Banks using donor screening, contamination control, and sterilization to reduce disease transmission, with the primary goal of providing surgeons with safe and effective bone tissue [14,37,49].

Xenografts are animal-derived bone substitutes and may originate from various sources, including bovine, porcine, equine, or coral exoskeletons. Their main advantages are easy availability and cost-effectiveness; however, they may transmit animal-derived infections, and their use can raise ethical and religious concerns [11,37,50]. Their composition is similar to that of human bone, with hydroxyapatite as the main component; therefore, they exhibit osteoconductive properties [37,50].

Alloplasts are synthetic materials that aim to create an alternative bone substitute similar to human bone [10,11]. They can be classified depending on their composition in metals, ceramics, polymers and composites [6]. These materials are cost-effective and do not carry the risk of disease transmission, are biocompatible, and are readily available, but their bone regeneration ability is inferior to that of autologous bone [9,13,40]. Synthetic bone substitutes have osteoconductive properties and can also be osteoinductive by adding BMPs during their production [10,36,39,40].

Allografts, as bone substitute materials, are widely employed across medical specialties—including orthopedics, traumatology, oral and maxillofacial surgery, neurosurgery, and otolaryngology—for diverse clinical indications such as fracture repair, degenerative musculoskeletal disorders, spinal arthrodesis, prosthetic revision, reconstructive procedures, and oncological surgery [1,2,5,16,17]. Allografts are also used in periodontology to treat bone and tissue defects, with BMP-containing grafts benefiting patients unresponsive to non-invasive therapies [13,37,46,48]. In oral and maxillofacial surgery, an adequate alveolar ridge—particularly in the esthetic zone—is crucial for achieving optimal implant-supported restorations [7,9,18,47,51]. Alveolar ridge augmentation increases the likelihood of successful osseointegration and implant survival and improves aesthetic outcomes [43,51,52]. In maxillofacial surgery, allografts can also be used in the treatment of craniofacial anomalies and bone defects resulting from trauma or malignancy [8,18,44].

The biological properties of allografts, including osteoconductive, osteoinductive, and osteogenic potential, are highly dependent on production and storage conditions [38]. The West Hungarian Regional Tissue Bank has over 30 years of experience in producing BMP-containing allografts, with excellent clinical outcomes in both orthopedic and oral surgery [18]. To ensure quality and safety, donors are selected under strict criteria and routinely screened for HIV-1/2, hepatitis B and C, and syphilis [5,16,45]. Bones can be classified by shape (long, short, flat, irregular) and structure (cortical or cancellous), with cortical bone—comprising ~80% of bone volume and rich in BMPs—being more metabolically active and preferable for BMP-containing allografts [6,18,38,46]. Before clinical use, allografts undergo multiple processing procedures [14]. The preparation process involves removing residual muscles, tendons, and ligaments from long bones, followed by cutting or fragmenting them. This step increases the efficiency of the defatting process [46]. The aim of this processing methodology is to enhance the bioavailability of the native BMPs present in cortical bone. Dilute hydrochloric acid, by mimicking osteoclast activity, facilitates the release of stored BMPs [4,14]. To minimize the risk of infectious disease transmission, allografts are subjected to deantigenization using chemical solutions and subsequent sterilization [3,14]. Gamma irradiation reduces the osteoinductive potential of grafts by degrading BMPs and is therefore not suitable for the production of BMP-containing allografts [3,40]. In contrast, ethylene oxide sterilization is an effective method that preserves BMP integrity [11]. Lyophilization further reduces antigenicity and, by removing approximately 95% of the bone’s water content, enables long-term storage of allografts at room temperature [14,46].

The aim of this article was to conduct a life cycle assessment of the manufacturing process of BMP-containing allografts under the baseline and six alternative scenarios in order to identify the environmental impacts and key hotspots of the process. The environmental footprint of healthcare includes various water, soil, and air pollutants, as well as negative impacts on human health [27]. Based on the results of the life cycle and sensitivity analyses, several potential interventions were identified to improve the environmental performance of the manufacturing process. The preservation of BMP is part of the process, but this LCA evaluates only the sustainability of production and does not assess the biological properties of the graft.

To assess environmental sustainability, it is necessary to use indicators that enable reporting, comparison, and clear demonstration of the outcomes of sustainability measures [25,26,30]. Measuring the carbon footprint, as a quantification of greenhouse gas emissions, is a widely used approach for this purpose [27,53]. However, the DALY metric aggregates the human health impacts of the ReCiPe midpoint categories into a single endpoint indicator, thereby enabling a more comprehensive assessment [23]. DALY values reflect the health burden associated with allograft production at the population level [54]. In this study, the DALY value of the BMG production process was 6.58 h, which may serve as a reference point for future research. The total DALY impact of producing 300 g of BMG (packaged in 1 g units) is 7.508 × 10^−4^. For comparison, a study assessing Hawley and Essix retainers reported total DALY values of 1.499 × 10^−7^ and 1.567 × 10^−7^ h per device, respectively, while another study on single-use and reusable flexible ureteroscopes (fURS) reported total DALY values of 4.57 × 10^−6^ and 1.15 × 10^−6^ per use, respectively. It should be noted that these studies report values per single use or device, whereas the BMG value corresponds to the production of 300 individual 1 g units. Considering these differences, the DALY impact of the BMG production process remains relatively low on a per-unit basis, but provides a meaningful reference for understanding the potential human health impacts associated with Tissue Bank operations [23,55].

Chemicals contribute substantially to environmental impacts across several impact categories, including climate change, and their replacement with alternatives associated with lower environmental burdens would therefore be desirable. However, the properties of allografts depend to a large extent on processing methods, including the types and quantities of chemicals used [12]. Several chemical processing methods are available, some of which can also be applied in combination [3,11,12]. Reducing the amount of chemical solutions may improve environmental performance; however, for instance, the concentration and volume of hydrochloric acid used during demineralization must be carefully controlled, as both insufficient and excessive treatment can adversely affect graft properties [14]. Further studies on chemical processing methods are therefore needed to identify approaches with more favorable environmental profiles that may also enhance the biological properties of allografts [12]. Chemical processing represents a critical trade-off between environmental sustainability and biological performance. In BMP-preserving allograft production, chemical agents are indispensable for ensuring defatting, deantigenization, and the bioavailability of native growth factors. While their contribution to environmental impacts is non-negligible, insufficient or overly aggressive chemical treatment may compromise graft quality and clinical effectiveness. Therefore, sustainability-oriented optimization should focus on refining chemical concentrations, exposure times, and recovery strategies rather than eliminating chemical steps.

The contribution analysis revealed that the environmental impacts of the chemical processing stage are mainly driven by a limited number of substances. In particular, the methanol–chloroform solution and hydrogen peroxide represent the major contributors across several impact categories, especially as climate change, acidification, and human toxicity indicators. Although PBS solution also shows relatively high contributions in several impact categories, these results should be interpreted with caution because a generic proxy dataset was used in the inventory; therefore, it was not considered a primary hotspot in the interpretation. Other chemicals (e.g., EDTA, hydrochloric acid, and calcium chloride) show comparatively minor contributions, while certain substances contribute more strongly only to specific categories. A detailed breakdown of the relative contributions of each chemical to the impact categories is provided in Supplementary Material S2.

Electricity use is also a major contributor to environmental impacts across several sustainability indicators in healthcare facilities [25]. Optimizing and reducing energy consumption would positively influence sustainability, as also indicated by the results of this life cycle assessment [25,30,56]. The integration of renewable energy sources into healthcare practice is receiving increasing attention [27,28]. Sensitivity analysis suggests that a transition to wind-generated electricity could substantially improve the environmental sustainability of allograft production and, indirectly, healthcare delivery.

Although allograft production involves a relatively limited amount of disposable materials, hospital activities generally require a high volume of single-use products [23,26]. Therefore, minimizing disposable materials or replacing them with biodegradable alternatives would represent a substantial step toward improving the environmental sustainability of healthcare [26,32]. Reusable versions of medical devices are associated with lower environmental impacts than their single-use counterparts [23,28]. For example, reusable surgical gowns and hair and shoe covers can reduce carbon footprints and operational costs without compromising infection control requirements [24,27]. Environmental responsibility also includes minimizing packaging and substituting it with more environmentally friendly alternatives [30,31]. Changing the packaging of allografts from 1 g to 5 g units may reduce environmental impacts; however, this must be considered in light of clinical practice. In oral surgery, such a large quantity is typically not required at once, which could increase material waste, whereas in orthopedic applications, larger quantities are often needed, making larger packaging units potentially more appropriate in that context. At the Tissue Bank, BMG is routinely packaged in 1 g units for oral surgery applications and 5 g units for orthopedic procedures, which was considered in the sensitivity analysis; clinical usage may vary, and larger amounts may be required, but these standard package sizes provide a practical reference for scaling the environmental impacts.

Waste management also contributes to environmental impacts. According to the sensitivity analysis, energy recovery from waste incineration could help make the production process more environmentally sustainable. To reduce waste in healthcare systems, regular waste audits should be conducted [20,25,26]. Proper segregation of hazardous and non-hazardous waste is essential for lowering the carbon footprint [28]. Additionally, reducing the use of disposable materials—for example, by employing reusable surgical gowns—can further decrease the volume of generated waste [30,56].

The scenarios modeled in the sensitivity analysis are not fully independent real-world cases but may provide guidance for making the production process more sustainable. According to the European Society of Intensive Care Medicine, sustainability measures can be grouped into three levels based on ease of implementation and environmental impact. Simple actions, such as reducing the use of single-use materials or switching off unused electrical equipment, are easy to implement, while measures like transitioning to renewable energy or conducting LCA of processes are more challenging but yield greater environmental benefits. Establishing green meetings and staff training is also necessary to promote sustainability awareness within the Tissue Bank [20].

LCA is complex and time-consuming; however, it has increasingly been investigated in healthcare activities [20]. According to Borglin et al., the carbon footprint of a hypothetical dental examination in a clinic is 0.73 kg CO_2_eq, and, in addition to the carbon footprint, land use and freshwater ecotoxicity are the main contributors to the overall environmental impacts [57]. Künzle et al. conducted an LCA of tooth extraction under two scenarios, resulting in a carbon footprint of 13.8 kg CO_2_eq when the consent form is signed in person (conventional consent process) and 8.8 kg CO_2_eq when it is signed online (digital consent process). In all of the cases listed, the LCA also includes patient travel, which, as reported in the literature, significantly influences the carbon footprint. In the case of tooth extraction, freshwater ecotoxicity, resource use, and land use are the primary contributors to environmental impacts [53,58]. For the manufacturing process of BMG bone substitutes, terrestrial ecotoxicity and the minerals and metals impact category are more relevant. For a meaningful comparison of carbon footprints between the two studies, it would be necessary to compare them with the LCA of bone augmentation procedures involving BMG, as both dental examination and tooth extraction involve the use of materials, and BMG can likewise be considered a biomaterial used in clinical procedures. Climate change is one of the greatest challenges facing healthcare [20]. Environmental sustainability refers to the implementation of measures that ensure access to resources for future generations [27,31,53,59]. Health systems should be designed to integrate environmental sustainability without compromising the quality of care [25]. All stakeholders involved in healthcare delivery contribute to the environmental impacts of healthcare systems [27,53]. To support informed decision-making, it is important to evaluate the roles of both manufacturers and distributors [21,32,60]. Green procurement represents a strategic approach to promote the development and adoption of more sustainable products and services [61,62].

The downstream processes were not included within the system boundaries; however, certain factors should still be considered when assessing potential environmental impacts. Attention should be given to implementing greener transportation from the point of manufacture to the point of use, as well as to adopting more environmentally sustainable practices in the operating room [26,30]. Surgical activities also contribute to global warming through resource consumption, with one study estimating that operating rooms in healthcare systems emit between 3000 and 5000 tonnes of greenhouse gases annually [22]. Prioritizing the use of sterilizable instruments, implementing selective waste segregation, and minimizing the use of anesthetic gases can help make surgical procedures more environmentally sustainable [22,24,26].

The activities of the West Hungarian Regional Tissue Bank include, among others, the production of allografts from femoral heads obtained from living donors. Femoral heads are rich in cancellous bone, and during their processing, no specific steps are taken to enhance BMP bioactivity; consequently, fewer chemical substances are used. The carbon footprint of these allografts is 100,332 kg CO_2_-eq per production cycle, compared with 66,759 kg CO_2_-eq for the production of BMP-containing, donor cadaver-derived bone substitutes (BMG). However, it should be taken into account that both products are packaged in 1 g units for oral implantology. On average, 500 g of femoral head-derived allografts and 300 g of BMG were produced per processing cycle. Accordingly, the carbon footprint allocated per unit is 0.200 kg CO_2_-eq for femur-derived allografts and 0.222 kg CO_2_-eq for BMG. When interpreting these results, it is important to consider that the system boundaries did not include immunogenicity testing of allografts in either case. The environmental impacts of such testing are likely to be higher for femur-derived bone substitutes, as bones from 24 individuals are processed in a single cycle, whereas in BMG production, a single donor cadaver is used, whose other tissues (e.g., heart, kidneys, skin) are also utilized for transplantation, requiring the environmental impacts to be allocated among multiple tissues. Based on these considerations, it is likely that the actual environmental impacts per 1 g unit are broadly comparable between the two production processes, although BMP-containing allografts appear more favorable from a clinical perspective. Nevertheless, donor availability should also be taken into account in the production of allografts from both sources [63].

Tissue engineering aims to induce the formation of new, functional bone tissue [39,64]. Tissue engineering approaches for the treatment of bone defects seek to mimic the natural process of bone healing; therefore, the essential components include cells, growth factors, and a supporting scaffold [6,39]. The scaffold, which may be composed of synthetic materials, provides the extracellular microenvironment necessary for cell-driven regeneration [15]. Among growth factors, BMP is one of the most potent osteogenic factors, plays a key role in tissue engineering, and can be derived from allografts [11,15]. Stem cells can be incorporated into in vitro-engineered grafts to generate bone tissue; however, this approach has not yet been approved for widespread clinical application [11]. Tissue engineering-based approaches may offer opportunities to improve the environmental sustainability of bone augmentation.

Although the literature on the environmental impacts of bone replacement interventions and materials is limited, some studies have evaluated the life cycle of tissue-engineered, 3D-printed bone substitutes using GaBi software. These studies report that the carbon footprint of 1 kg of bone scaffolds produced from different raw materials ranges between 1335 and 3.07 kg CO_2_-eq, with electricity identified as a major contributor to environmental impacts [65,66,67]. Based on these findings, tissue engineering appears to be a promising approach for improving the environmental sustainability of bone replacement; however, further comparative studies are required to enable a more comprehensive and accurate assessment.

### Limitations of the Study

The study has several limitations that may affect the interpretation of the results. During the life cycle assessment, conversion factors specific to Hungary were not always available. These factors were derived from older datasets, and the values may have changed since their collection. Another limitation is the absence of immunogenicity testing, the allocation of which would have been methodologically uncertain due to the diverse applications of multiple donor tissues. Furthermore, the exclusion of downstream processes represents an additional limitation, and further studies may be required to analyze the full life cycle from cradle to grave of allograft use. Finally, due to the limited availability of literature data, the results could not be fully compared with the environmental impacts of other bone substitute materials; nevertheless, this study could provide a foundation for future research in this area.

## 5. Conclusions

The climate change impact of the production of a donor cadaver-derived, BMP-containing granular bone substitute allograft was 66.759 kg CO_2_-eq. Chemicals used in the process contributed the most to this impact, while electricity consumption also represented a significant contribution across several impact categories. The use of renewable energy sources (e.g., wind power) could substantially improve the environmental performance of allograft production. Precise and controlled use of chemicals is essential for preserving BMPs and maintaining the biological properties of the bone substitute for clinical application. Therefore, it is important to identify chemical agents that support BMP preservation and facilitate defatting and deantigenization, while exhibiting lower environmental impacts.

## Figures and Tables

**Table 1:** Technical performance of the equipment used during allograft production.

Nr	Device	Model	Performance
1	Fridge/Freezer	Midea Chest Freezer HS-543CN, Midea Group, China, Beijao	333 W
2	Bone-cleaning machine	N/A	350 W
3	Bone cutting machine	N/A	1500 W
4	Bone grinding machine	Ikaweke, IKA-Werke GmbH&Co. KG, Germany, Staufen	1000 W
5	Sieve machine	Retsch AS 200, Retsch GmbH, Germany, Haan	315 W
6	Thermostat	L MIM	5500 W
7	Ethylen oxide sterilizer	Steri/Vac 5XL Gas Sterilizer, 3M Company, USA, Minneapolis, MN	2300 W
8	Sealer machine	Steriking RS 3200, Wipak, Finland, Helsinki	240 W
9	Lyophilizer	Scanvac Coolsafe 90-80 Superior, LaboGene A/S, Denmark, Allerød	2100 W
10	Air conditioner	AUX, AUX Group, China, Ningbo	5400 W
11	Laptop	Lenovo E1 Vision, Lenovo Group Limited, China, Beijing	15 W
12	Laminar box	Laminar Box Airflow BPV-1200 FRM, Radel&Hahn zrt, Hungary, Debrecen	750 W
13	Autoclave	N/A	100 W

**Table 2:** Life cycle impact results: The total burden on the environment caused by one production process of allografts (300 g): on-demand calculation type.

Impact Category	Reference Unit	Results
Acidification: terrestrial (TAP)	kg SO_2_-Eq	3.576
Climate change (GWP)	kg CO_2_-Eq	6.675 × 10^1^
Ecotoxicity: freshwater (FETP)	kg 1,4-DCB-Eq	4.927 × 10^−1^
Ecotoxicity: marine (METP)	kg 1,4-DCB-Eq	8.029 × 10^−1^
Ecotoxicity: terrestrial (TETP)	kg 1,4-DCB-Eq	2.637 × 10^2^
Energy resources: non-renewable, fossil (FFP)	kg oil-Eq	2.922
Eutrophication: freshwater (FEP)	kg P-Eq	3.840 × 10^−3^
Eutrophication: marine (MEP)	kg N-Eq	6.460 × 10^−4^
Human toxicity: carcinogenic (HTPc)	kg 1,4-DCB-Eq	1.081
Human toxicity: non-carcinogenic (HTPnc)	kg 1,4-DCB-Eq	2.836 × 10^1^
Ionizing radiation (IRP)	kBq Co-60-Eq	1.848 × 10^1^
Land use (LOP)	m^2^*a crop-Eq	1.658 × 10^−1^
Material resources: metals/minerals (SOP)	kg Cu-Eq	4.613 × 10^2^
Ozone depletion (ODP)	kg CFC-11-Eq	2.772 × 10^−4^
Particulate matter formation (PMFP)	kg PM_2.5_-Eq	1.080
Photochemical oxidant formation: human health (HOFP)	kg NOx-Eq	2.702 × 10^−1^
Photochemical oxidant formation: terrestrial ecosystems (EOFP)	kg NOx-Eq	2.759 × 10^−1^
Water use (WCP)	m^3^	5.022 × 10^−3^

**Table 3:** Results of DALY values.

DALYs	Results
Climate change: human health	6.195 × 10^−5^
Human toxicity: carcinogenic	3.590 × 10^−6^
Human toxicity: non-carcinogenic	6.466 × 10^−6^
Ionizing radiation	1.570 × 10^−7^
Ozone depletion	1.471 × 10^−7^
Particulate matter formation	6.783 × 10^−4^
Photochemical oxidant formation: human health	2.459 × 10^−7^
Water use: human health	1.115 × 10^−8^
Total:	7.508 × 10^−4^
Total in days:	2.742 × 10^−1^
Total in hours:	6.582

## Data Availability

Data presented are available upon request from the corresponding author.

## Supplementary Materials

The following supporting information can be downloaded at https://www.mdpi.com/article/10.3390/jfb17040171/s1. Supplementary Materials S1: Life Cycle Inventory; Supplementary Materials S2: Results of Uncertainty and Sensitivity Analysis.

## Abbreviations

The following abbreviations are used in this manuscript:

LCA	Life Cycle Assessment
ISO	International Organization for Standardization
CO_2_	carbon dioxide
LCI	Life Cycle Inventory
LCIA	Life Cycle Impact Assessment
HIV	Human Immunodeficiency Virus
GHG	greenhouse gases
DALY	disability-adjusted life years
BMP	bone morphogenetic protein
PDGF	platelet-derived growth factor
VEGF	vascular endothelial growth factor
FGF	fibroblast growth factor
HLA	human leukocyte antigen
BMG	bone matrix gelatin
