# Supplementary material for: Towards Sustainable Bone Grafting: Life Cycle Assessment of Donor Cadaver-Derived Allograft (BMG) Production Using a BMP-Preserving Approach
Source: J Funct Biomater. 2026 Apr 1;17(4):171. doi: 10.3390/jfb17040171 (PMC13117126; doi:10.3390/jfb17040171)
Supplement: Supplementary file 1 [file jfb-17-00171-s001.zip › jfb-4174539-supplementary.pdf]

Supplementary Material 2 – Uncertainity and sensitivity analysis

1. Uncertainity analysis

Table 1.1.: Results of Monte Carlo simulations

Impact category	Reference unit	Mean	Standard deviation	Minimum	Maximum	Median	5% Percentile	95% Percentile
**TAP**	kg SO2-Eq	3,580E+00	9,109E-01	1,635E+00	9,394E+00	3,459E+00	2,325E+00	5,220E+00
**GWP**	kg CO2-Eq	6,683E+01	7,835E+00	4,597E+01	1,056E+02	6,604E+01	5,546E+01	8,117E+01
**FETP**	kg 1,4-DCB-Eq	4,954E-01	2,331E-01	1,545E-01	3,313E+00	4,440E-01	2,435E-01	9,028E-01
**METP**	kg 1,4-DCB-Eq	8,068E-01	3,203E-01	3,258E-01	4,826E+00	7,356E-01	4,635E-01	1,371E+00
**TETP**	kg 1,4-DCB-Eq	2,643E+02	4,196E+01	1,671E+02	5,268E+02	2,595E+02	2,060E+02	3,393E+02
**FFP**	kg oil-Eq	2,932E+00	6,516E-01	1,310E+00	6,744E+00	2,859E+00	2,008E+00	4,122E+00
**FEP**	kg P-Eq	3,882E-03	2,103E-03	8,718E-04	4,468E-02	3,418E-03	1,798E-03	7,362E-03
**MEP**	kg N-Eq	6,466E-04	1,775E-04	3,087E-04	2,125E-03	6,154E-04	4,305E-04	9,760E-04
**HTPc**	kg 1,4-DCB-Eq	1,089E+00	6,242E-01	3,815E-01	1,612E+01	9,431E-01	5,905E-01	2,014E+00
**HTPnc**	kg 1,4-DCB-Eq	2,847E+01	1,058E+01	1,238E+01	2,603E+02	2,653E+01	1,782E+01	4,471E+01
**IRP**	kBq Co-60-Eq	1,851E+01	5,337E+00	7,646E+00	6,324E+01	1,773E+01	1,170E+01	2,772E+01
**LOP**	m2*a crop-Eq	1,660E-01	4,818E-02	6,153E-02	4,368E-01	1,596E-01	1,016E-01	2,569E-01
**SOP**	kg Cu-Eq	4,618E+02	1,240E+02	1,983E+02	1,250E+03	4,450E+02	2,906E+02	6,857E+02
**ODP**	kg CFC-11-Eq	2,777E-04	4,925E-05	1,541E-04	5,339E-04	2,720E-04	2,062E-04	3,638E-04
**PMFP**	kg PM2.5-Eq	1,082E+00	2,780E-01	4,879E-01	2,856E+00	1,045E+00	6,986E-01	1,582E+00
**HOFP**	kg NOx-Eq	2,705E-01	3,463E-02	1,791E-01	4,344E-01	2,667E-01	2,207E-01	3,348E-01
**EOFP**	kg NOx-Eq	2,762E-01	3,491E-02	1,840E-01	4,388E-01	2,723E-01	2,261E-01	3,408E-01
**WCP**	m3	5,024E-03	1,743E-03	1,507E-03	1,743E-02	4,714E-03	2,782E-03	8,230E-03

2. Sensitivity analysis

Table 2.1.: Results of Sensitivity analysis in midpoint categories

Impact category	Reference unit	Base scenario	Conventional chemical	Chemical -20	Green energy	Energy -20	Energy recovery from waste	5g packages
**TAP**	kg SO2-Eq	3,576E+00	3,570E+00	3,561E+00	1,989E-01	3,561E+00	3,574E+00	3,540E+00
**GWP**	kg CO2-Eq	6,676E+01	6,620E+01	6,110E+01	6,406E+01	6,110E+01	6,159E+01	5,770E+01
**FETP**	kg 1,4-DCB-Eq	4,928E-01	4,780E-01	4,551E-01	4,911E-01	4,551E-01	2,189E-01	4,090E-01
**METP**	kg 1,4-DCB-Eq	8,029E-01	7,830E-01	7,475E-01	7,243E-01	7,475E-01	4,380E-01	6,800E-01
**TETP**	kg 1,4-DCB-Eq	2,637E+02	2,620E+02	2,587E+02	1,207E+02	2,587E+02	2,605E+02	2,560E+02
**FFP**	kg oil-Eq	2,923E+00	2,750E+00	2,535E+00	2,923E+00	2,535E+00	2,472E+00	2,050E+00
**FEP**	kg P-Eq	3,841E-03	3,640E-03	3,365E-03	3,841E-03	3,365E-03	2,555E-03	2,760E-03
**MEP**	kg N-Eq	6,461E-04	6,020E-04	6,008E-04	6,443E-04	6,008E-04	3,234E-04	4,960E-04
**HTPc**	kg 1,4-DCB-Eq	1,082E+00	1,060E+00	1,003E+00	9,224E-01	1,003E+00	6,914E-01	9,100E-01
**HTPnc**	kg 1,4-DCB-Eq	2,837E+01	2,780E+01	2,694E+01	1,508E+01	2,694E+01	2,194E+01	2,520E+01
**IRP**	kBq Co-60-Eq	1,848E+01	1,840E+01	1,810E+01	2,457E+00	1,810E+01	1,812E+01	1,760E+01
**LOP**	m2*a crop-Eq	1,659E-01	1,570E-01	1,379E-01	1,659E-01	1,379E-01	1,450E-01	1,050E-01
**SOP**	kg Cu-Eq	4,613E+02	4,610E+02	4,613E+02	1,408E+00	4,613E+02	4,613E+02	4,610E+02
**ODP**	kg CFC-11-Eq	2,772E-04	2,770E-04	2,561E-04	1,477E-04	2,561E-04	2,763E-04	2,420E-04
**PMFP**	kg PM2.5-Eq	1,081E+00	1,080E+00	1,076E+00	4,953E-02	1,076E+00	1,080E+00	1,070E+00
**HOFP**	kg NOx-Eq	2,703E-01	2,690E-01	2,448E-01	2,633E-01	2,448E-01	2,676E-01	2,240E-01
**EOFP**	kg NOx-Eq	2,759E-01	2,750E-01	2,503E-01	2,647E-01	2,503E-01	2,730E-01	2,290E-01
**WCP**	m3	5,023E-03	4,340E-03	4,209E-03	5,023E-03	4,209E-03	4,283E-03	2,990E-03

Table 2.2.: Results of Sensitivity analysis in DALY endpoint category

DALYs	Climate change: human health	Human toxicity: carcinogenic	Human toxicity: non-carcinogenic	Ionising radiation	Ozone depletion	Particulate matter formation	Photochemical oxidant formation: human health	Water use: human health	Total
**Conventional chemical use**	6,142E-05	3,504E-06	6,334E-06	1,559E-07	1,471E-07	6,780E-04	2,452E-07	9,645E-09	7,498E-04
**Reduced chemical use (-20%)**	5,670E-05	3,329E-06	6,140E-06	1,537E-07	1,360E-07	6,755E-04	2,227E-07	9,343E-09	7,422E-04
**Energy supplied from wind-power**	5,945E-05	3,062E-06	3,440E-06	2,084E-08	7,841E-08	3,114E-05	2,396E-07	1,115E-08	9,745E-05
**Reduced energy demand (-20%)**	6,144E-05	3,482E-06	5,851E-06	1,297E-07	1,332E-07	5,486E-04	2,446E-07	1,115E-08	6,199E-04
**Energy recovery from waste incineration**	5,716E-05	2,295E-06	5,000E-06	1,539E-07	1,467E-07	6,777E-04	2,435E-07	9,509E-09	7,427E-04
**Use of 5g packaging units**	5,352E-05	3,020E-06	5,735E-06	1,497E-07	1,285E-07	6,721E-04	2,038E-07	6,630E-09	7,349E-04

3. Environmental impacts of the individual chemicals used during processing

Table 3.1.: Results of environmental impacts of the individual chemicals used during processing

**Chemical substances**	**Metanol-chloroform solution**	**Hydrogen peroxide**	**Acetic acid**	**PBS solution**	**Calcium chloride**	**EDTA**	**Hydrochloric acid**	**Litium chloride**	**Deionised water**	**Ethylene oxid**
**TAP**	44,77%	9,29%	0,01%	41,92%	0,97%	0,71%	0,06%	0,60%	0,01%	1,67%
**GWP**	50,23%	21,00%	0,01%	23,76%	0,31%	1,19%	0,59%	0,41%	0,01%	3,01%
**FETP**	0,08%	0,01%	0,00%	86,46%	1,77%	2,87%	0,50%	1,97%	0,05%	6,30%
**METP**	0,28%	0,30%	0,00%	86,39%	1,71%	2,79%	0,48%	1,92%	0,05%	6,07%
**TETP**	5,26%	18,67%	0,00%	66,86%	1,05%	2,19%	0,38%	1,40%	0,03%	4,16%
**FFP**	0,00%	0,00%	0,00%	71,54%	0,73%	4,81%	0,20%	1,05%	0,02%	21,65%
**FEP**	0,00%	0,00%	0,00%	87,20%	1,81%	2,66%	0,58%	2,92%	0,07%	4,80%
**MEP**	0,31%	0,46%	0,00%	75,81%	1,35%	2,37%	0,46%	14,49%	0,06%	3,13%
**HTPc**	2,20%	0,07%	0,00%	88,31%	1,12%	2,87%	0,29%	2,13%	0,03%	2,99%
**HTPnc**	0,34%	1,13%	0,00%	84,22%	1,96%	2,95%	0,56%	2,11%	0,06%	6,67%
**IRP**	0,00%	0,00%	0,00%	87,60%	0,73%	3,43%	0,83%	1,40%	0,15%	94,13%
**LOP**	0,00%	0,00%	0,00%	90,07%	1,49%	2,19%	0,49%	1,92%	0,11%	3,74%
**SOP**	0,00%	0,00%	0,00%	11,62%	0,11%	0,27%	0,04%	87,44%	0,01%	0,50%
**ODP**	28,31%	10,98%	0,00%	56,65%	0,02%	0,04%	0,01%	0,03%	0,00%	0,10%
**PMFP**	44,78%	9,31%	0,01%	41,72%	0,82%	0,78%	0,07%	0,56%	0,01%	1,94%
**HOFP**	73,27%	15,23%	0,01%	9,88%	0,16%	0,31%	0,02%	0,17%	0,00%	0,96%
**EOFP**	72,83%	15,14%	0,01%	10,29%	0,16%	0,34%	0,02%	0,18%	0,00%	1,04%
**WCP**	0,00%	0,00%	0,00%	79,21%	1,56%	3,04%	0,39%	10,75%	0,05%	4,91%
